# Time-series trends and regional disparities in complete denture production in Brazil (2000–2023): an epidemiological analysis

**DOI:** 10.1590/1678-7765-2025-0779

**Published:** 2026-06-19

**Authors:** Maria Eduarda Broering da Silva, Henrique Souza dos Santos, Juliana Silva Ribeiro de Andrade, Paulo Roberto Barbato, Maurício Malheiros Badaró

**Affiliations:** 1 Universidade Federal de Santa Catarina Departamento de Odontologia Florianópolis Brasil Universidade Federal de Santa Catarina (UFSC), Departamento de Odontologia, Florianópolis, Brasil.; 2 Universidade Federal de Santa Catarina Departamento de Fonoaudiologia Florianópolis Brasil Universidade Federal de Santa Catarina (UFSC), Departamento de Fonoaudiologia, Florianópolis, Brasil.

**Keywords:** Complete denture, Interrupted time series analysis, Public health dentistry, Health services accessibility, Epidemiology

## Abstract

**Introduction:**

Tooth loss remains a public health problem in Brazil, reflecting social inequality, population aging, and limited access to oral rehabilitation. For many individuals, complete dentures (CD) are the only option available free of charge through the Brazilian Unified Health System (Sistema Único de Saúde, SUS). However, nationwide assessments of service delivery and regional equity in prosthetic rehabilitation are limited.

**Objective:**

To quantify national trends and regional disparities in CD production within the SUS and examine how prosthetic output relates to demographic, socioeconomic, and infrastructure indicators using the most recent data available.

**Methodology:**

This ecological-analytical time-series study used open data from the SUS Outpatient Information System (SIA/SUS). The analysis included maxillary and mandibular CD procedures recorded between 2000 and 2022, representing the most recent consolidated period for temporal modeling, and a state-level snapshot for 2023 to describe the latest correlational scenario. Production rates were standardized per 100,000 inhabitants. Temporal trends were analyzed using segmented linear regression to estimate the average annual percent change (AAPC), which summarizes overall long-term changes in complete denture production, and to identify inflection points. Correlations between CD production and demographic or socioeconomic indicators were tested using Spearman’s rho (α = 0.05).

**Results:**

Between 2000 and 2022, a total of 5,239,089 complete dentures were delivered through the Brazilian Unified Health System. CD production increased significantly nationwide from 2000 to 2022 (AAPC = 9.7%; 95% CI: 4.8–14.9), a relatively high annual growth rate for public health service expansion, with the steepest rise between 2006 and 2014 (AAPC = 29.2%; 95% CI: 18.6–40.7). Regional trends varied across the country, with the highest growth observed in the Northeast (AAPC = 16.4%; 95% CI: 11.7–21.3), followed by the North (AAPC = 14.3%; 95% CI: 10.9–17.7), Central-West (AAPC = 13.4%; 95% CI: 4.9–22.5), and South (AAPC = 12.8%; 95% CI: 9.0–16.7), whereas the Southeast showed the smallest expansion (AAPC = 8.0%; 95% CI: −1.7–18.8). In 2023, the SUS network included 2,903 prosthetic laboratories, highlighting the organizational infrastructure supporting prosthetic rehabilitation services. In 2023, São Paulo (Southeast region) produced 111,396 dentures, and Piauí (Northeast region) recorded the highest production rate (833.36 per 100,000 inhabitants). Strong correlations were observed between CD production and population size (ρ = 0.742, p < 0.001) and number of municipalities (ρ = 0.938, p < 0.001).

**Conclusion:**

Over the past two decades, complete denture production within the SUS expanded substantially, reflecting the consolidation of Brazil’s public oral health policies. However, persistent regional disparities and heterogeneous production rates indicate that service expansion has not translated into equitable coverage nationwide. These findings highlight the importance of demographic and organizational factors in shaping prosthetic service provision and underscore the need for targeted planning and resource allocation to reduce inequalities in access to oral rehabilitation.

## Graphical abstract

**Figure:**
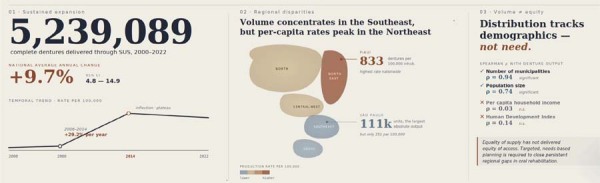

## Introduction

Edentulism remains a major public health problem worldwide and continues to disproportionately affect socially vulnerable populations and older adults.^1,2^ In Brazil, tooth loss reflects the cumulative effects of lifelong exposure to oral diseases, limited access to preventive care, and persistent socioeconomic inequalities.^3,4^ Despite improvements in the epidemiological profile of oral health, edentulism remains a significant burden for the Brazilian health system, sustaining demand for prosthetic rehabilitation within the Unified Health System (SUS).^2^

Before 2000, the provision of prosthetic rehabilitation within the SUS was limited, fragmented, and largely dependent on local initiatives, with little national coordination and restricted availability of specialized infrastructure.^5^ Prosthetic care was not yet systematically incorporated into oral health policies, resulting in low and uneven service coverage across Brazilian regions.^6^ This scenario began to change with the strengthening of public oral health policies, culminating in the implementation of the National Oral Health Policy (*Brasil Sorridente*) in 2004, which reorganized oral health care, expanded access to specialized services, and incorporated prosthetic rehabilitation into a structured national framework.^7,8,9^ National population-based surveys have played a central role in documenting the magnitude and distribution of edentulism in Brazil.^10^

The SB Brasil 2023 survey, published in 2025, documented the distribution of tooth loss and prosthetic needs across Brazilian regions, particularly among older adults, providing robust cross-sectional estimates of oral health conditions and unmet needs at the population level.^11^ Such national oral health surveys constitute an essential reference for public health planning.^12^ However, due to their cross-sectional design, these surveys do not capture how prosthetic service provision evolves over time, nor do they allow assessment of long-term trends, regional trajectories, or temporal inflection points related to policy implementation, system expansion, or external shocks. Consequently, important aspects of the dynamics of prosthetic service delivery within the Unified Health System (SUS) remain insufficiently explored.

In this context, data from the Outpatient Information System of the Unified Health System (SIA/SUS) enable the evaluation of prosthetic service production over extended periods and allow the identification of temporal trends and regional disparities in access to care, complementing evidence generated by population-based surveys.^13,14^ The originality of this study lies in its national time series spanning more than two decades, encompassing periods before and after major policy milestones and the COVID-19 pandemic.

Furthermore, the application of segmented linear regression (Joinpoint analysis) enables the formal identification of inflection points and changes in growth patterns over time. Therefore, this study aimed to analyze temporal trends in complete denture production within the Brazilian Unified Health System from 2000 to 2022 and to assess regional and state-level disparities using the most recent 2023 cross-sectional data. By integrating time-series analysis with demographic, socioeconomic, and service-related indicators, this study complements population-based surveys and provides evidence relevant to oral health policy evaluation and planning. These findings may provide valuable evidence to guide health managers and policymakers in optimizing the planning and equitable distribution of prosthetic rehabilitation services nationwide. The null hypothesis was that no significant temporal changes in complete denture production or associations with contextual indicators would be observed.

## Methodology

### Study design

This study employed an observational, ecological, analytical time-series design, based on secondary data extracted from official open-access health information systems. Data were obtained from the Outpatient Information System of the Brazilian Unified Health System (Sistema Único de Saúde, SUS), which records all outpatient procedures performed nationwide. The study involved no direct participation of human subjects, and all analyses were conducted using aggregated, publicly available data. Therefore, ethical approval was not required, in accordance with the Brazilian National Health Council Resolution No. 510/2016.^15^ This study was conducted and reported in accordance with the Strengthening the Reporting of Observational Studies in Epidemiology (STROBE) guidelines.^16^

### Data sources and variables

Complete denture production data were extracted from the Outpatient Information System of the Brazilian Unified Health System (SIA/SUS; DATASUS, Brasília, DF, Brazil; https://datasus.saude.gov.br), which compiles nationwide records of outpatient dental procedures. The dataset included all maxillary and mandibular complete denture fabrication procedures recorded between 2000 and 2023, the most recent year available in the system. Although the SIA/SUS database provides separate records for maxillary and mandibular complete dentures, these procedures were aggregated to represent overall prosthetic service provision and ensure consistent temporal and regional comparisons. For temporal trend modeling, only data from 2000 to 2022 were included, as these years represent the most recent period with fully consolidated and validated records suitable for time-series analysis. Data from 2023 were excluded from temporal modeling due to ongoing validation but were retained for cross-sectional descriptive and correlational analyses. Procedures unrelated to new complete denture fabrication, such as relining and repairs (SIGTAP 0307040089) or removable partial dentures (maxillary SIGTAP 0701070102 and mandibular SIGTAP 0701070099), were excluded. The data were aggregated by year and by state (n = 27). All datasets were accessed in September 2025 from publicly available DATASUS repositories.

Data on the number and classification of Dental Specialty Centers (DSCs; Types I, II, and III) and the Regional Dental Prosthesis Laboratories (RPLs) were retrieved from the Brazilian Ministry of Health (Brasília, DF, Brazil; https://www.gov.br/saude), under the *Brasil Sorridente* initiative, according to the criteria established in Ordinance No. 599/2006.^17^ Dental Specialty Centers (DSCs) are classified into Types I, II, and III according to structural capacity and number of dental chairs, reflecting increasing service complexity. This classification was used to characterize the distribution and potential capacity of specialized oral health care across Brazilian states.^17^ The most recent consolidated information, referring to 2023, was used to represent the current structure of prosthetic service provision in each state.

Demographic and socioeconomic indicators—including total population, population density, number of municipalities, mean household income per capita, and the Human Development Index (HDI)—were obtained from the Brazilian Institute of Geography and Statistics (IBGE; Rio de Janeiro, RJ, Brazil; https://www.ibge.gov.br ). The selection of these variables was grounded in health services research frameworks, in which demographic characteristics reflect potential service demand and territorial organization, socioeconomic indicators capture structural inequalities influencing access to care, and administrative indicators such as the number of municipalities represent the complexity of service organization and resource allocation within decentralized public health systems. All indicators were compiled at the state level (n = 27) to enable correlation analyses with complete denture production.

The dependent variables were: (1) the absolute number of complete dentures produced, calculated as the annual sum of maxillary and mandibular dentures recorded in the SIA/SUS database; and (2) the complete denture production rate per 100,000 inhabitants, computed according to the formula: Rate (state,year) = Number of dentures (state,year) / Population (state,year) ×100,000. Population data were obtained from the 2000, 2010, and 2022 national censuses and intercensal estimates provided by the IBGE.

The independent variables comprised: (1) Demographic and socioeconomic indicators: total population, population density (inhabitants/km^2^), number of municipalities, mean household income per capita (Brazilian reais), and the Human Development Index (HDI); and (2) Service infrastructure indicators: number and classification (Types I, II, and III) of Dental Specialty Centers (DSCs) and the number of Regional Dental Prosthesis Laboratories (RPLs) in each state.

### Data processing and statistical analysis

Data extraction and compilation were independently conducted by two trained operators (MEDBS and HSS) using publicly available DATASUS and IBGE databases. Both operators followed identical, predefined extraction procedures and inclusion criteria. The resulting datasets were compiled into standardized spreadsheets and cross-checked for consistency and accuracy before statistical analysis. Data were organized and tabulated using Microsoft Excel 2016 (Microsoft Corporation, Redmond, WA, USA) for descriptive analysis and generation of tables and figures. The normality of data distribution was verified using the Shapiro–Wilk test. As variables did not follow a normal distribution (p< 0.05), correlations between demographic, socioeconomic, and service-related indicators were assessed using Spearman’s rank correlation coefficient (ρ). The strength of correlations was interpreted according to commonly adopted thresholds: |ρ| < 0.30 as weak, |ρ| = 0.30–0.59 as moderate, and |ρ| ≥ 0.60 as strong.

All inferential analyses were performed in IBM SPSS Statistics for Windows, Version 25.0 (IBM Corp., Armonk, NY, USA), adopting a significance level of α = 0.05. Temporal trends in complete denture production between 2000 and 2022 were examined using segmented linear regression through the Joinpoint Regression Program, Version 4.5.0.1 (Statistical Research and Applications Branch, National Cancer Institute, Bethesda, MD, USA). This method allows identification of joinpoints (inflection points) and estimation of Annual Percent Change (AAPC) values with corresponding 95% confidence intervals (95% CI), indicating statistically significant shifts in trend direction. In the segmented linear regression analysis, time intervals were not predefined. Joinpoints (inflection points) were identified only when statistically significant changes in trend were detected. Consequently, the number and duration of temporal segments varied across regions according to the presence of significant joinpoints, while regions without significant inflection points were modeled using a single segment covering the entire study period.

Descriptive results were summarized as median, minimum, and maximum values. Graphical visualizations were generated to illustrate the geographic distribution of prosthetic infrastructure and the rate of denture production per 100,000 inhabitants across Brazilian states.

### Data availability

All datasets and analytical codes used in this study are publicly accessible through the SciELO Data Repository (São Paulo, SP, Brazil) under DOI: 10.48331/SCIELODATA.K8EHUT . The repository contains aggregated, anonymized data originally derived from the DATASUS platform of the Brazilian Ministry of Health, ensuring full transparency, traceability, and reproducibility of the analyses.

### Ethical compliance statement

This study used secondary, open-access data obtained from official public databases of the Brazilian Ministry of Health and involved no direct participation of human subjects. According to the Brazilian National Health Council Resolution No. 510/2016 (Article 1, Item III)^15^ research based exclusively on publicly available, non-identifiable data is exempt from Research Ethics Committee review. Therefore, ethics approval was not required for this investigation.

## Results

**Figure 1:**
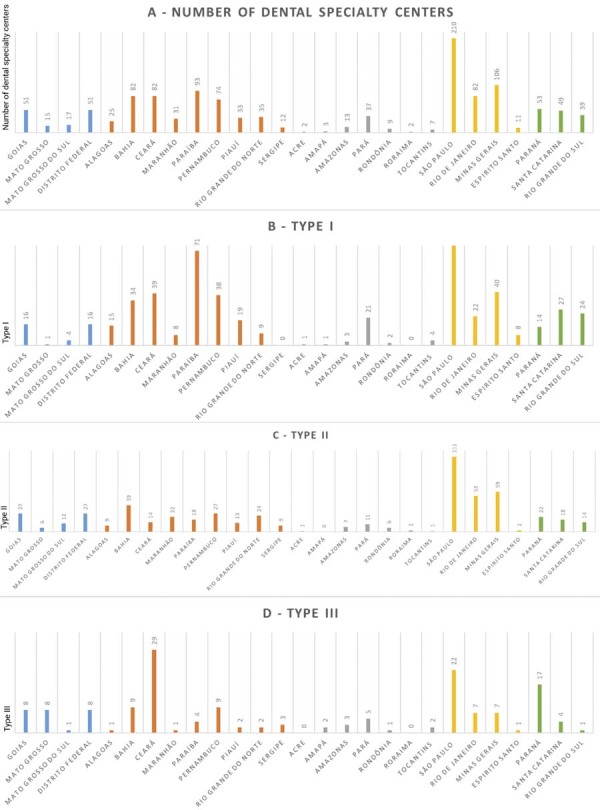
– Distribution of Dental Specialty Centers (DSCs) in Brazilian states, 2023. (A) Total number of DSCs; (B) Type I centers; (C) Type II centers; (D) Type III centers. DSC = Dental Specialty Center. Bars are color-coded by geographic region: Central-West (blue), Northeast (orange), North (gray), Southeast (yellow), and South (green). Absolute values are displayed above each bar.

In the 2023 cross-sectional analysis, Table 1 presents the distribution of variables according to tertiles of Dental Specialty Centers (DSCs). The number of DSCs was categorized into three groups: up to 15 (n = 9), 16 to 51 (n = 10), and 52 or more (n = 8) centers. As the number of DSCs increased, there was a consistent rise in the number of Regional Dental Prosthesis Laboratories (RPLs) and in the total production of complete dentures, indicating a positive association between service infrastructure and prosthetic output. Figure 1 illustrates the geographic distribution of DSCs by type (I, II, and III) across Brazilian states in 2023. São Paulo recorded the highest number of DSCs (210 centers), whereas Acre and Roraima registered the lowest counts, with only two centers each.

**Table 1:** Distribution of service infrastructure and complete denture production according to tertiles of Dental Specialty Centers (DSCs), Brazil, 2023.

Variable	DSCs per state (tertiles)	Median	Minimum	Maximum	Total values
DSCs Type I (n)	Up to 15	1	0	8	20
	16 to 51	16	4	27	159
	52 or more	39	14	77	335
DSCs Type II (n)	Up to 15	2	0	9	34
	16 to 51	16	9	27	177
	52 or more	33	14	111	343
DSCs Type III (n)	Up to 15	2	0	8	20
	16 to 51	2	1	8	33
	52 or more	9	4	29	104
Regional Dental Prosthesis Laboratories (RPLs, n)	Up to 15	22	4	85	254
	16 to 51	124	1	185	1.100
	52 or more	151	47	444	1.549
Complete denture production (units)	Up to 15	3,988	140	10,374	32.172
	16 to 51	17,527	69	28,385	168.179
	52 or more	31,374	6,854	111,396	360.136

DSCs: Dental Specialty Centers.

**Figure 2:**
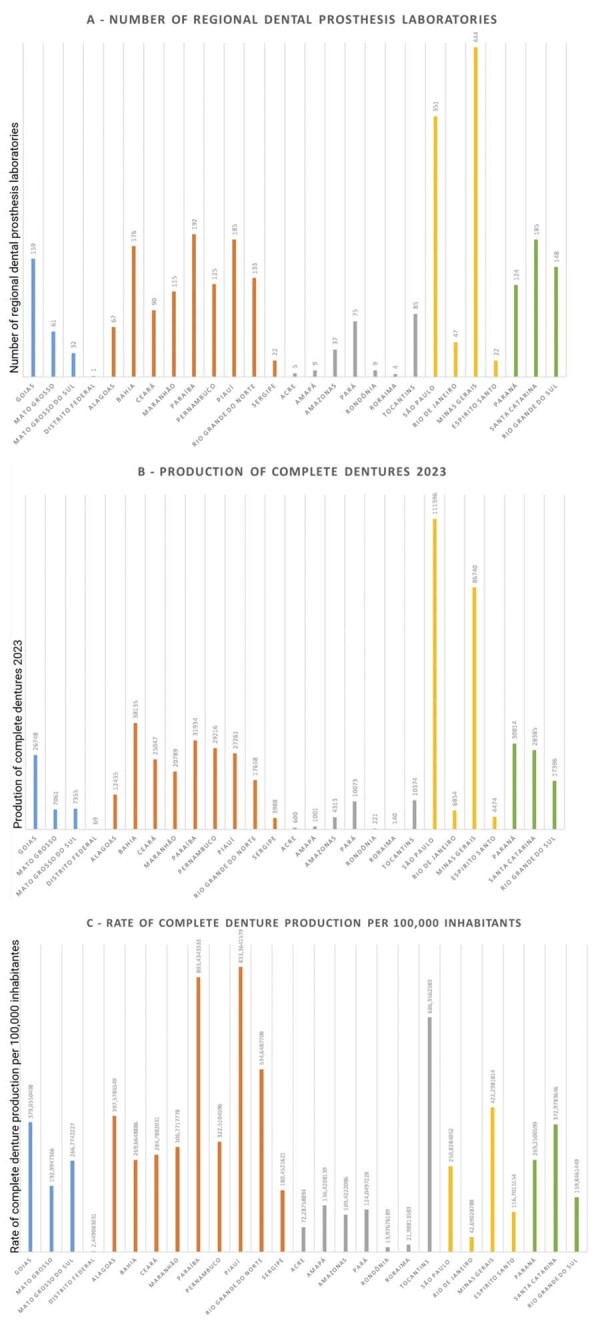
– Regional Dental Prosthesis Laboratories (RPLs) and complete denture production by state, 2023.

Regarding the 2023 cross-sectional analysis, Figure 2 displays the number of Regional Dental Prosthesis Laboratories (RPLs), the total production of complete dentures, and the rate of denture production per 100,000 inhabitants across Brazilian states. Minas Gerais had the highest number of RPLs (444 laboratories), whereas São Paulo showed the largest total denture production (111,396 units). In contrast, Piauí recorded the highest production rate relative to population size, with 833.36 complete dentures per 100,000 inhabitants, compared with 250.83 per 100,000 inhabitants in São Paulo, despite São Paulo presenting the largest absolute production (111,396 units versus 27,261 in Piauí).

(A) Number of RPLs; (B) Total number of complete dentures produced; (C) Production rate per 100,000 inhabitants. Bars are color-coded by geographic region: Central-West (blue), Northeast (orange), North (gray), Southeast (yellow), and South (green). Absolute values are displayed above each bar.

Correlation analyses (Table 2) demonstrated statistically significant associations between the number of Dental Specialty Centers (DSCs) and population size (ρ = 0.86; p < 0.001), as well as the number of municipalities (ρ = 0.73; p < 0.001). Similar significant correlations with population size and number of municipalities were observed for DSC Type I (ρ = 0.80 and 0.74, respectively; both p < 0.001), DSC Type II (ρ = 0.79 and 0.65, respectively; both p < 0.001), and DSC Type III (ρ = 0.67 and 0.47, respectively; p < 0.001 and p = 0.012). The number of DSCs was also positively correlated with population density (ρ = 0.59; p = 0.001), as were DSC Type I (ρ = 0.55; p = 0.003) and DSC Type II (ρ = 0.60; p = 0.001), whereas the association for DSC Type III did not reach statistical significance (ρ = 0.37; p = 0.053). No statistically significant association was observed between the number of DSCs and the Human Development Index (HDI) (ρ = 0.37; p = 0.052). Among DSC subtypes, only Type II showed a significant correlation with HDI (ρ = 0.39; p = 0.042). Additionally, the number of Regional Dental Prosthesis Laboratories (RPLs) and complete denture production were significantly associated with population size (ρ = 0.70 and 0.74, respectively; both p < 0.001) and number of municipalities (ρ = 0.95 and 0.93, respectively; both p < 0.001).

**Table 2:** Spearman correlations between state-level indicators and prosthetic service infrastructure/output (DSCs, RPLs, complete denture production), Brazil, 2023.

Variable	Population density		Per capita household income		Human Development Index (HDI)		Number of municipalities		Population	
	ρ	p-value	ρ	p-value	ρ	p-value	ρ	p-value	ρ	p-value
Number of Dental Specialty Centers (DSCs)	0.597	**0.001**	0.268	0.177	0.378	0.052	0.730	**<0.001**	0.867	**<0.001**
DSC Type I	0.553	**0.003**	0.179	0.371	0.307	0.120	0.744	**<0.001**	0.808	**<0.001**
DSC Type II	0.607	**0.001**	0.296	0.134	0.393	**0.042**	0.655	**<0.001**	0.790	**<0.001**
DSC Type III	0.376	0.053	0.245	0.217	0.361	0.064	0.477	**0.012**	0.673	**<0.001**
Number of Regional Dental Prosthesis Laboratories (RPLs)	0.256	0.197	0.102	0.614	0.149	0.459	0.950	**<0.001**	0.704	**<0.001**
Complete denture production (units)	0.308	0.118	0.026	0.899	0.139	0.489	0.938	**<0.001**	0.742	**<0.001**
Production rate of complete dentures per 100,000 inhabitants	0.138	0.493	-0.173	0.389	-0.131	0.516	0.64	**<0.001**	0.22	0.269

Notes: ρ = Spearman correlation; n = 27 states; HDI = Human Development Index; DSC: Dental Specialty Center.

**Figure 3:**
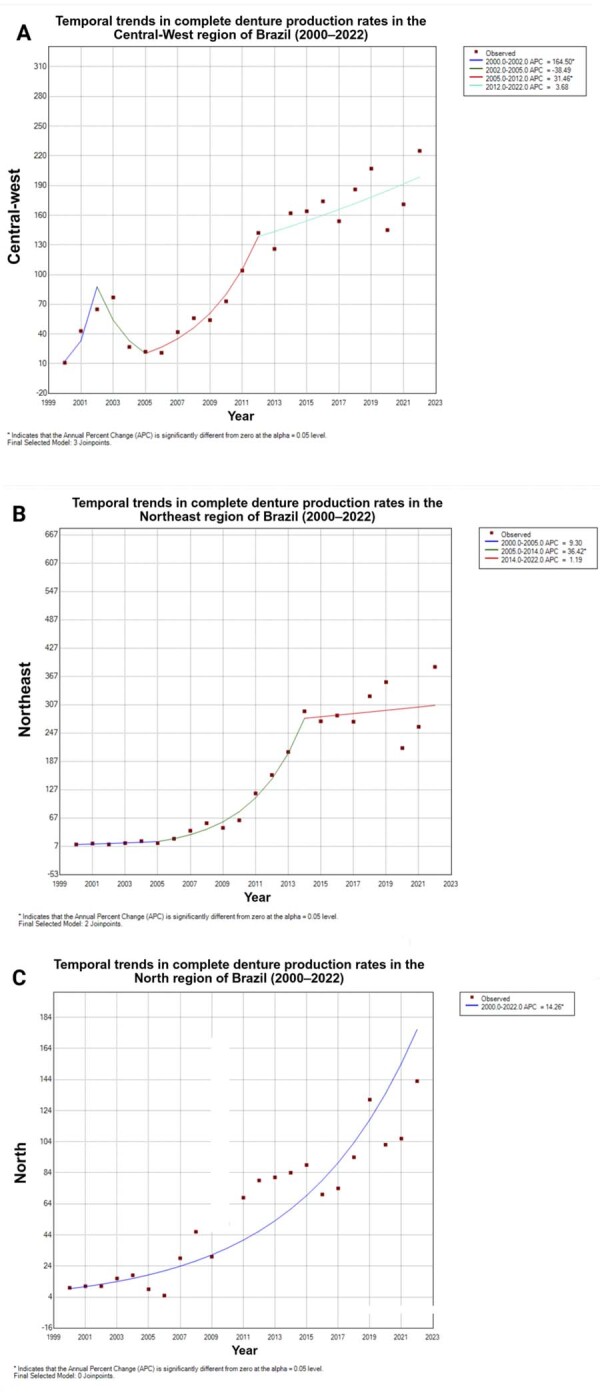
– Average annual percent change (AAPC) in complete denture production rates in regions of Brazil, 2000–2022: (A) Central-West, (B) Northeast, (C) North.

**Figure 4:**
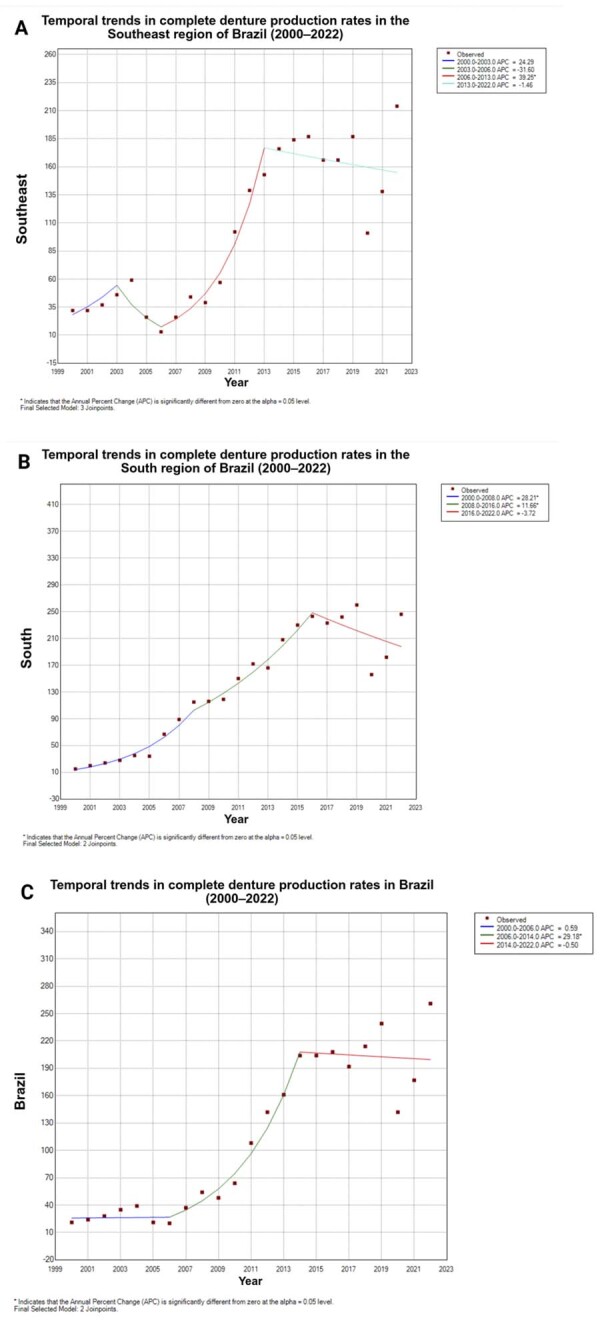
– Average annual percentage variation in the complete denture production rate in Brazil and regions from 2000 to 2022: (A) Southeast, (B) South, (C) Brazil.

In the 2000–2022 time-series analysis, Table 3 summarizes the average annual percent change (AAPC) in complete denture production rates in Brazil and its major regions. Nationwide and across all regions (Figures 3 and 4), production rates showed a significant upward trend throughout the study period. The national AAPC was 9.7% (95% CI: 4.8–14.9), ranging from 12.8% (95% CI: 9.0–16.7) in the South to 16.4% (95% CI: 11.7–21.3) in the Northeast. The steepest national growth occurred between 2006 and 2014, with an AAPC of 29.2% (95% CI: 18.6–40.7). In the Central-West, significant increases were observed during 2000–2002 and 2005–2012. In the Northeast, the sharpest rise occurred from 2005 to 2014, while in the Southeast, a marked increase was detected between 2006 and 2013. The South region exhibited two distinct phases of growth (2000–2008 and 2008–2016), whereas no segmented trend was identified in the North during the analyzed period.

**Table 3:** Average annual percent change (AAPC) in complete denture production rates, Brazil and regions, 2000–2022.

Region	Segment	Start year	End year	AAPC (%)	95%CI		p-value
Brazil	Total	2000	2022	9.7*	4.8	14.9	<0.05
	1	2000	2006	0.6	-9.7	12.0	0.9
	2	2006	2014	29.2*	18.6	40.7	**<0.05**
	3	2014	2022	-0.5	-7.2	6.7	0.9
Central-west region	Total	2000	2022	13.4*	4.9	22.5	**<0.05**
	1	2000	2002	164.5*	62.0	331.8	**<0.05**
	2	2002	2005	-38.5	-62.3	0.4	0.1
	3	2005	2012	31.5*	21.0	42.8	**<0.05**
	4	2012	2022	3.7	-0.2	7.7	0.1
Northeast region	Total	2000	2022	16.4*	11.7	21.3	**<0.05**
	1	2000	2005	9.3	-3.8	24.2	0.2
	2	2005	2014	36.4*	28.2	45.2	**<0.05**
	3	2014	2022	1.2	-4.9	7.7	0.7
North region	Total	2000	2022	14.3*	10.9	17.7	**<0.05**
Southeast region	Total	2000	2022	8.0	-1.7	18.8	0.1
	1	2000	2003	24.3	-9.5	70.7	0.2
	2	2003	2006	-31.6	-63.7	29.0	0.2
	3	2006	2013	39.2*	25.1	55.0	**<0.05**
	4	2013	2022	-1.5	-7.0	4.4	0.6
South region	Total	2000	2022	12.8*	9.0	16.7	**<0.05**
	1	2000	2008	28.2*	21.8	35.0	**<0.05**
	2	2008	2016	11.7*	4.8	19.0	<0.05
	3	2016	2022	-3.7	-11.1	4.3	0.3

AAPC = Average annual percent change; *Statistically significant AAPC. CI = confidence interval; APC = annual percent change for individual segments; Statistically significant at α = 0.05.

Overall, the findings reveal a continuous national increase in complete denture production between 2000 and 2022, with distinct regional growth patterns and no segmented trend in the North. The 2023 cross-sectional data confirm that states with a greater number of Dental Specialty Centers (DSCs) also tend to have more Regional Dental Prosthesis Laboratories (RPLs) and higher prosthetic output, although per-capita rates vary considerably across regions. Together, these results demonstrate an overall expansion of prosthetic service provision within the Unified Health System, alongside persistent regional disparities in infrastructure and access. Higher concentrations of Dental Specialty Centers and Regional Dental Prosthesis Laboratories were observed in the Southeast and South regions, particularly in states such as São Paulo and Minas Gerais, whereas lower availability of specialized services was evident in the North and parts of the Central-West, where states such as Acre and Roraima present the lowest infrastructure levels.

## Discussion

This study demonstrated a sustained national increase in complete denture production within the Brazilian Unified Health System (SUS), accompanied by marked regional heterogeneity and ecological associations with service infrastructure and demographic factors. The study hypothesis was rejected, as a sustained national increase in complete denture production was observed over the last two decades, accompanied by strong ecological associations with population size, number of municipalities, and the distribution of oral health service infrastructure. Rather than reflecting isolated service performance, these findings reveal structural patterns in the organization and expansion of prosthetic rehabilitation within Brazil’s public oral health care network.

Within the Brazilian public oral health system, Dental Specialty Centers (DSCs) and Regional Dental Prosthesis Laboratories (RPLs) function as structural references for organizing prosthetic rehabilitation across the care network.^18,19^ Although complete removable dentures may be delivered at different levels of care, the associations observed between service infrastructure and denture production should be interpreted as indicating system-level capacity, coordination, and logistical support rather than the direct attribution of prosthetic fabrication to secondary care.^8,20-22^

The 2023 cross-sectional analysis demonstrated that states with larger populations and a greater number of municipalities tended to concentrate more DSCs, RPLs, and higher absolute volumes of complete denture production.^13,23^ São Paulo recorded the highest number of DSCs and the largest total prosthetic output, reflecting its demographic scale and extensive service network.^24^ In contrast, Piauí exhibited the highest production rate per 100,000 inhabitants, a finding that may be interpreted considering its smaller population size, high accumulated prosthetic needs, and historical prioritization of oral health service expansion in the Northeast region.^25^ Importantly, no direct inference can be made regarding specific local policies, investments, or municipal management initiatives, as such factors were not directly assessed in this study. Instead, this pattern likely reflects broader system-level characteristics and demographic denominators rather than discrete policy actions.^26^

Population size, number of municipalities, and population density showed significant ecological associations with the distribution of DSCs and RPLs, reinforcing the role of demographic and administrative factors in shaping health service infrastructure.^20,27^ In Brazil’s decentralized health system, municipal fragmentation may improve geographic proximity to services,^28^ while simultaneously increasing the complexity of organizing and sustaining specialized care, particularly in regions with limited technical and financial capacity.^29-31^ These dynamics help explain why infrastructure and service output tend to cluster in more populous or administratively complex states.

Notably, no statistically significant association was observed between complete denture production or the number of DSCs and socioeconomic indicators such as per capita income or the Human Development Index (HDI). This absence of correlation constitutes an important finding. It suggests that the allocation of prosthetic services within the SUS is more strongly influenced by demographic and organizational factors than by socioeconomic development alone.^14,32^ While this may reflect the universal and non-means-tested design of the Brazilian health system, it also suggests limitations in prioritizing areas with greater social vulnerability and higher unmet needs.^12,33^ In this context, equality in service distribution does not necessarily translate into equity, particularly in regions where socioeconomic disadvantage and prosthetic demand are disproportionately high.^34,35^

From a temporal perspective, complete denture production increased significantly nationwide between 2000 and 2022, with the most pronounced growth occurring between 2006 and 2014. This phase coincides with the consolidation of the National Oral Health Policy, increased federal investment in oral health, and rapid expansion of prosthetic services within the SUS.^8,30,36^ The observed growth also reflects accumulated demand resulting from historical tooth loss and population aging, particularly among older adults with limited prior access to preventive care.^13,37^

However, the Joinpoint analysis identified a clear inflection point around 2014, marking the end of the period of accelerated expansion. The deceleration observed from 2014 onward cannot be attributed solely to the COVID-19 pandemic, which began in Brazil in 2020. Instead, this trend likely reflects a combination of factors, including partial stabilization of prosthetic service expansion following the reduction of accumulated demand in some regions, constraints in health system financing, and broader economic challenges.^38^ The implementation of fiscal austerity measures and changes in federal health funding policies, such as the public spending cap established by Constitutional Amendment No. 95, may have limited the sustained expansion of oral health infrastructure and service capacity.^39,40^ The COVID-19 pandemic should therefore be interpreted as an aggravating factor that further disrupted elective dental services in the final years of the series, rather than as the primary cause of the observed trend break.^13,41^

Regional analyses revealed heterogeneous growth patterns that further underscore structural differences in service organization. The Northeast exhibited the highest overall growth, associated with its historically low baseline, high prosthetic needs, and targeted expansion of oral health services following national policy initiatives.^42,43^ In the North, production increased steadily without identifiable inflection points, suggesting gradual and continuous expansion shaped by territorial dispersion, low population density, and logistical challenges that favor incremental rather than abrupt service growth.^13^ In contrast, the South and Southeast regions, where oral health infrastructure is more consolidated, showed slower relative growth and earlier stabilization, consistent with partial saturation of accumulated demand.^44^

Data from SB Brasil 2023 reinforce the interpretation of a persistent mismatch between prosthetic needs and service provision. The highest prevalence of edentulism and need for complete dentures was observed in the North and Northeast, particularly among older adults, whereas service infrastructure and production capacity remain unevenly distributed.^45,46^ When considered alongside the present findings, these data indicate that increased production alone has not been sufficient to eliminate regional inequalities in access to oral rehabilitation.^12,13,42^

Similar challenges in the organization and coverage of oral health services have also been reported in other Latin American countries.^47^ Comparative analyses indicate that oral health policies in the region have evolved within distinct health system models, which directly influence access to dental services and prosthetic rehabilitation.^47^ Brazil represents a universal model of health coverage through the Unified Health System (SUS), where oral health is incorporated as part of a publicly financed national policy.^48^ In contrast, Chile operates under a dual public–private model, characterized by a segmented structure in which the public system (FONASA) coexists with private insurance schemes (ISAPRES), often resulting in stratified access to dental care.^49^ Colombia, in turn, follows a pluralistic insurance-based model with regulated competition among providers, where coverage depends on contributory or subsidized insurance regimes.^50,51^ These structural differences across health systems influence the availability and organization of oral health services, frequently resulting in uneven coverage and persistent inequalities in access to dental care across Latin America.^47,50^ Despite these variations, studies consistently report that oral health services in the region tend to prioritize specific population groups—such as children, pregnant women, or vulnerable populations—while comprehensive prosthetic rehabilitation services remain limited within public health systems.^52,53^

The ecological nature of this study must be explicitly acknowledged. All analyses were conducted using aggregated state-level data derived from independent administrative and demographic databases. There is no direct linkage between the geographic location of denture production, the place of residence of individuals, or population density. Consequently, the observed associations represent ecological inferences that indicate broader structural and organizational patterns of the health system rather than direct or causal relationships. Additionally, potential geographic mismatches between service location, production capacity, and population demand, including intermunicipal and interstate patient flows, may influence the interpretation of infrastructure-related findings.^20,54^

In addition to these considerations, the use of administrative health databases such as SIA/SUS may introduce specific sources of bias and imprecision. These systems are primarily designed for service registration and reimbursement rather than research purposes, variations in data recording practices across states or over time may influence the completeness and accuracy of procedure reporting. Underreporting or delays in data consolidation could potentially lead to underestimation of prosthetic production in certain years or regions. Moreover, analytical decisions adopted in this study, such as the aggregation of maxillary and mandibular complete dentures and the use of state-level ecological indicators, may influence the interpretation of results by emphasizing overall service output rather than individual treatment patterns. Although these choices were made to ensure consistent national comparisons and to reflect the organization of public prosthetic services, they may contribute to imprecision in estimating localized patterns of care. Future studies using SUS administrative data could benefit from integrating additional data sources, such as individual-level clinical records, municipal-level indicators, or longitudinal patient tracking systems, in order to improve the precision of estimates and allow more detailed assessments of access, service utilization, and continuity of prosthetic rehabilitation within the public health system.

From a planning perspective, the geographic concentration of prosthetic laboratories observed in this study may prompt reflections on alternative models of service organization within the SUS. Without extending beyond the scope of the present analysis, emerging strategies such as improved coordination between levels of care, regionalized planning, and the potential use of digital workflows that streamline design and production processes may represent future avenues to enhance access and efficiency, particularly in geographically extensive or underserved regions.^21,43,55^

Finally, while denture production is an important indicator of access to prosthetic rehabilitation, it represents only one component of comprehensive care. Complete dentures are static devices supported by biologically dynamic tissues, and continuity of care, including follow-up, adjustment, and maintenance, is essential for long-term effectiveness.^56^ Limited access to ongoing care may compromise functional outcomes and patient satisfaction, particularly in vulnerable populations.^57,58^ Future studies and policy planning should therefore consider not only the volume of prosthetic output but also the organization of longitudinal care within the public health system.

Despite its limitations, this study demonstrates that Brazil’s public oral health policies have substantially expanded access to prosthetic rehabilitation over the past two decades. However, persistent regional inequalities and the observed deceleration in service expansion highlight the need for renewed investment, strategic planning, and equity-oriented policies to address accumulated demand and ensure comprehensive oral rehabilitation within the SUS.

## Conclusion

Over the past two decades, complete denture production within the SUS expanded substantially, reflecting the consolidation of Brazil’s public oral health policies. However, persistent regional disparities and heterogeneous production rates indicate that service expansion has not translated into equitable coverage nationwide. These findings highlight the importance of demographic and organizational factors in shaping prosthetic service provision and underscore the need for targeted planning and resource allocation to reduce inequalities in access to oral rehabilitation.
